# Label-free functional and structural imaging of liver microvascular complex in mice by Jones matrix optical coherence tomography

**DOI:** 10.1038/s41598-021-98909-6

**Published:** 2021-10-08

**Authors:** Pradipta Mukherjee, Arata Miyazawa, Shinichi Fukuda, Toshiharu Yamashita, Donny Lukmanto, Kosuke Okada, Ibrahim Abd El-Sadek, Lida Zhu, Shuichi Makita, Tetsuro Oshika, Yoshiaki Yasuno

**Affiliations:** 1 Computational Optics Group, University of Tsukuba, Tsukuba, Ibaraki Japan; 2 Department of Ophthalmology, Faculty of Medicine, University of Tsukuba, Tsukuba, Ibaraki Japan; 3 Department of Advanced Vision Science, Faculty of Medicine, University of Tsukuba, Tsukuba, Ibaraki Japan; 4 Laboratory of Regenerative Medicine and Stem Cell Biology, Graduate School of Comprehensive Human Sciences, University of Tsukuba, Tsukuba, Ibaraki Japan; 5 Division of Medical Sciences, Faculty of Medicine, University of Tsukuba, Tsukuba, Ibaraki Japan; 6 Department of Physics, Faculty of Science, Damietta University, 34517 New Damietta City, Damietta Egypt

**Keywords:** Liver, Liver diseases, Microscopy, Imaging and sensing

## Abstract

We demonstrate label-free imaging of the functional and structural properties of microvascular complex in mice liver. The imaging was performed by a custom-built Jones-matrix based polarization sensitive optical coherence tomography (JM-OCT), which is capable of measuring tissue’s attenuation coefficient, birefringence, and tiny tissue dynamics. Two longitudinal studies comprising a healthy liver and an early fibrotic liver model were performed. In the healthy liver, we observed distinctive high dynamics beneath the vessel at the initial time point (0 h) and reappearance of high dynamics at 32-h time point. In the early fibrotic liver model, we observed high dynamics signal that reveals a clear network vascular structure by volume rendering. Longitudinal time-course imaging showed that these high dynamics signals faded and decreased over time.

## Introduction

The liver is regarded as the central metabolic organ of the body and is responsible for maintaining whole-body homeostasis^1^. The high metabolic activity in the liver is supported by an efficient microvasculature and the surrounding tissues. The microvasculature and surrounding tissues play significant roles in providing oxygen and nutrients to the different organs, promoting metabolic waste removal, and enabling the rapid response of the immune system^2^.

Physiological and structural changes in the liver microvasculature and the surrounding tissues, which we call the microvascular complex here, are associated with several types of liver disease that can advance to liver cirrhosis and ultimately to hepatocellular carcinoma (HCC)^3,4^. Liver inflammation is considered to be the initial stage of liver tissue damage and excessive damage can lead to liver fibrosis^5–9^. Therefore, assessment and longitudinal monitoring of the functional and structural properties of the liver’s microvascular complex are essential for detection of such liver diseases. In addition, the microvasculature has a three-dimensional (3D) network architecture. Therefore, a noninvasive 3D imaging modality that is sensitive to the tissue’s metabolism would serve as a perfect modality for investigation of the microvascular complex.

Histological evaluation of the liver tissue is the conventional method used to observe the microvascular complex and the cellular organization of a liver lobule^10,11^. However, this method is destructive and only allows a specific 2D cross-section of the vascular architecture and the tissue microstructure to be observed. Widely used imaging modalities, including computed tomography^12^, magnetic resonance imaging (MRI)^13^, and ultrasound^14,15^, can reveal the 3D microvascular complex architecture. However, computed tomography does not have any sensitivity to tissue function. In addition, both MRI and ultrasound can only offer poor resolution and thus cannot visualize the micron-scale structure of the microvascular complex. Fluorescence microscopy is another suitable method for investigation of the microvascular complex^16^, but it frequently requires use of labeling agents. This technique is thus invasive and is not the perfect modality for longitudinal tissue evaluation. None of the techniques above satisfy all the requirements, i.e., for nondestructive, noninvasive, and functional imaging.

Optical coherence tomography (OCT) is a noninvasive imaging method and can produce high resolution 3D images of biological tissues^17^. Extensions to OCT, including Doppler OCT and OCT angiography (OCTA), are sensitive to both tissue functions and functional structures and have been used in blood flow imaging^18–20^ and microvasculature imaging^21–24^, respectively. However, these methods do not have sensitivity to the tissue metabolism.

Recently, OCT-based dynamics imaging techniques have emerged that enable label-free, noninvasive depth-resolved investigation of tissue activity^25–31^. These dynamics imaging methods use several different techniques, including time-frequency analysis of the OCT signal^25,29,30^, the temporal variance of the time-sequence OCT signal^28^, and the correlation decay speed of the OCT signal^28,31^, and they then visualize the intra-cellular motion on a pixel-by-pixel basis. OCT-based dynamics imaging was originally performed using time-domain full-field OCT^25,26^, and was recently demonstrated using high-speed spectral-domain and swept-source OCT-based systems^28–30^. This approach has been used to investigate various tissue types, including ex vivo murine organs^25,29^, *in vitro* tumor spheroids^28^, human biopsy samples^30^, and in vivo human retina^31^.

The tissue microstructure can be investigated using the attenuation coefficient of OCT. It is the attenuation speed of the OCT signal along the depth direction. Attenuation coefficient imaging has been used in several applications including investigations of tumor spheroid necrosis^32,33^, atherosclerotic plaque characterization^34^, glaucoma diagnosis and monitoring^35^, investigation of ocular tissue properties^36^, and examination of coronary artery tissues^37^.

The tissue microstructure can also be assessed via polarization-sensitive (PS-) OCT imaging^38^, which includes use of local phase retardation or equivalently birefringence^39–43^. PS-OCT is sensitive to collagenous microstructures, e.g., fibrotic tissues, and it has been used to investigate fibrous atherosclerotic plaques in the human aorta^44,45^, cancerous tissues^46^, and retinal fibrosis^47,48^.

In this paper, we demonstrate tissue function imaging of the ex vivo mouse liver microvascular complex using our newly developed OCT-based dynamics imaging method, “logarithmic intensity variance (LIV)”^28^. The LIV is the time variance of the logarithmic OCT signal and the LIV is sensitive to intra-cellular motion within the tissue. In addition, attenuation coefficient (AC) and birefringence images were acquired simultaneously along with the LIV image. This simultaneous multi-contrast imaging method was performed using our custom-made Jones-matrix-based polarization-sensitive swept-source OCT. Two different studies were organized on ex vivo freshly dissected mouse liver tissues. The first study involved longitudinal imaging of an ex vivo healthy mouse liver every 6 h. The second study involved 3D imaging of the ex vivo early fibrotic mouse liver model. The mice were treated with carbon-tetrachloride (CCl_4_) continuously for two days for the early fibrotic liver model. The microvasculature complex was imaged every 4 h.

## Results

### Imaging of healthy mouse liver at the initial time point

**Figure 1:**
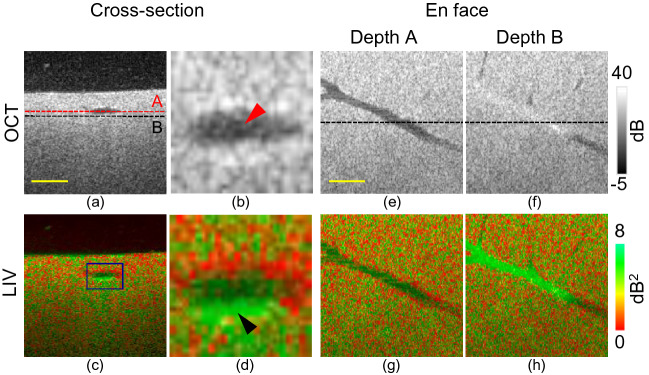
Dynamics imaging of healthy mouse liver at the initial (0 h) time point. (**a**, **c**) Cross-sections from scattering OCT and LIV imaging; (**b**, **d**) magnified images of the cross-sectional images at the region indicated by the rectangular box in (**c**); (**e**, **g**) *en face* slices of OCT and LIV images at the depth location indicated by the red horizontal line in (**a**); (**f**, **h**) *en face* slices at the depth location indicated by the black horizontal line in (**a**). Scale bar: 250 μm.

Figure 1 summarizes the cross-sectional and *en face* OCT intensity and LIV images of the healthy mouse liver 1 h after sacrification ; this point is denoted as the initial time point or 0 h. Volumetric imaging was performed, and the cross-sectional images were then extracted from the volumetric data. Figure 1e–h show the *en face* OCT and LIV slices at the two depth positions (A and B) indicated by the red and black horizontal lines shown in Fig. 1a, respectively. Figure 1b and d are magnified images of the boxes shown in Fig. 1a and c. The black spaces at the top of the cross-sectional images (Fig. 1a and c) represent the cultured medium, and the small white spots present in the medium in Fig. 1a represent floaters in the medium.

The OCT intensity image of the healthy mouse liver is homogeneous. The lumen appears as a hyposcattering region in the cross-sectional OCT image (red arrowhead, Fig. 1b). This low-scattering lumen corresponds to the vessel structure that can be observed in the *en face* OCT image at depth A. Just beneath the lumen (depth B), the OCT signal shows relatively high scattering (Fig. 1f).

The cross-sectional LIV image shows high LIV (green) just beneath the lumen (black arrowhead, Fig. 1d). This high LIV indicates high temporal fluctuation in the OCT signal and hence it may indicate high intra-cellular motion. The *en face* LIV image at depth B, i.e., just below the vessel, shows high LIV (green) and its shape is analogous to that of the vessel (Fig. 1h). This appearance of high LIV just beneath the vessel indicates that the inter-cellular motion around the vessel wall at the initial time point is high. The same experiment was performed on another four healthy mouse liver samples using the same measurement protocol and similar results to those in Fig. 1 were obtained (see Supplementary Material).

In addition to LIV imaging, Jones-matrix OCT (JM-OCT)^49,50^ can provide the OCT intensity, an attenuation coefficient (AC) image, and a birefringence image from a single measurement. A synthesized tissue contrast is then obtained by applying principal component analysis (PCA) to the LIV, AC, and birefringence images.

**Figure 2:**
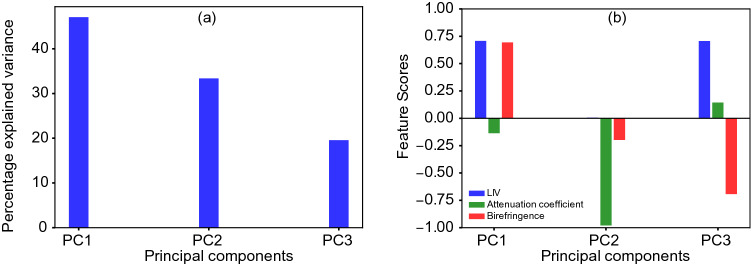
(**a**) Percentage variances of the three principal components and (**b**) contributions of each feature (feature scores) to the corresponding principal components for the healthy mouse liver at the zero-hour time point. PC: principal component.

The percentage variance results for the principal components (PCs) and the feature scores are shown in Fig. 2. As shown in Fig. 2a, the first PC (PC1) has approximately 50% explained variance, but both PC2 and PC3 also have significant explained variance percentages. As shown in Fig. 2b, the feature scores of the LIV and the birefringence correspond positively to each other with the same magnitude in PC1. PC2 is contributed almost entirely by the AC. Similar to PC1, the LIV and the birefringence are almost equal in PC3. However, in contrast to the case in PC1, they correspond negatively to each other.

**Figure 3:**
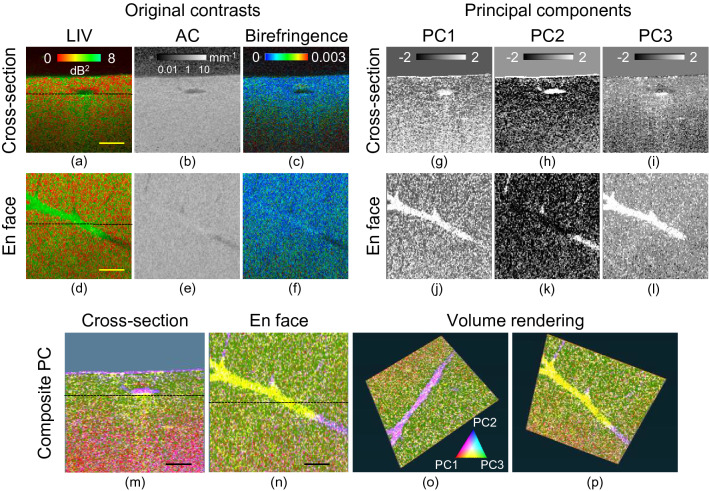
Principal component analysis of healthy mouse liver at 0 h time point. (**a**–**f**) Multi-contrast cross-sectional and *en face* images of the input features, including AC, LIV, and birefringence. (**g**–**l**) Cross-sectional and *en face* images of the principal components (PCs). (**m**–**p**) The composite PC (bottom row) is generated by assigning the red channel to PC1, the blue channel to PC2, and the green channel to PC3. Scale bar: 250 μm.

Figure 3a–f represent the cross-sectional and *en face* images of the LIV, AC, and birefringence. From these images, images of PC1 to PC3 were generated, as shown in Fig. 3g–l. The depth locations of the *en face* slices and the locations of the B-scans in the *en face* images are indicated by the horizontal lines in Fig. 3a and d, respectively. A pseudo-color PC image was generated by assigning PC1, PC2, and PC3 to the red, blue, and green channels, respectively (Fig. 3).

The maximum variances in PC1 and PC3 were observed just beneath the vessel (yellow), which means that the maximum variations from the mean values of the three contrasts mostly occur beneath the vessel. In contrast, the maximum variance for PC2 was observed within the vessel. Because of the collocation of PC1, it appears in purple. In all the PCs, the nonvessel regions show relatively lower variance compared with the vessel surrounding tissue regions (i.e., the microvascular complex) and are almost homogeneous.

The pseudo-color images (Fig. 3m–p) give characteristic appearances to some of the tissue structures. The surfaces of the tissue and the vessel appear in magenta, which represents the combined contributions of PC1 and PC2. The tissue just beneath the vessel appears in yellow, which represents the mixed contributions of PC1 and PC3. The higher depth regions appear in red, while the moderate depth regions appear in green. Although the appearance of the OCT intensity image is mostly homogeneous, the pseudo-color PC images reveal distinctive structures.

### Time-lapse imaging of healthy liver for longitudinal evaluation

**Figure 4:**
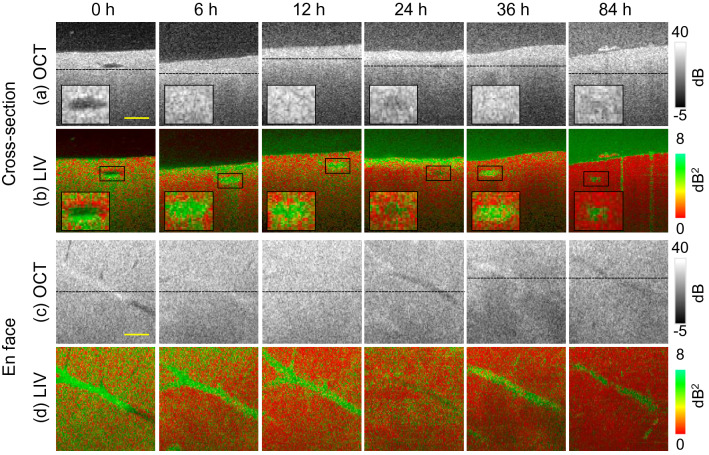
Longitudinal dynamics visualization of healthy liver microvasculature. The first and second rows (**a**, **b**) show cross-sectional and the third and fourth rows (**c**, **d**) show *en face* slices of the OCT and LIV images, respectively. The first to fourth rows show the time-course images at the 6, 12, 24, 36, and 84 h time points. Scale bar: 250 μm.

To validate the activity of the microvascular complex, longitudinal measurement was performed at the 0 (initial time point), 6, 12, 24, 36, and 84 h time points, as shown in Fig. 4. Note that the 0 h time point image is identical to that of Fig. 1. The first and second rows (Fig. 4a and b) show the time-course cross-sectional images of OCT and LIV, while the third and fourth rows (Fig. 4c and d) represent the *en face* OCT and LIV images. The depth locations of these *en face* slices are indicated by the horizontal lines in the corresponding B-scan OCT images.

The vessel lumen, which appeared as the hyposcattering region at the 0 h time point in the OCT signal (Fig. 1a), cannot be observed at the 6 h and 12 h time points in the OCT intensity images. At these time points, the OCT signal at the suspected vessel region shows higher scattering relative to that shown in Fig. 1a. This may be caused by contraction or expansion of the sample, which was observed during the experiments.

At the 24 h time point, we see that the vessel structure appears again as a relatively low-scattering region in the magnified cross-sectional and *en face* OCT images. At the 36 h time point, the OCT signal shows relatively high scattering within the vessel region, whereas at the later time points, e.g., at 84 h, it appears again as a relatively low scattering region.

The cross-sectional and *en face* LIV images (second and fourth rows, respectively) show that, at the 6 h and 12 h time points, the OCT signal fluctuation magnitudes in the microvascular complex are still large (green), while they are low (red ) in the nonvessel regions. At later time points, e.g., at 24 h, the high LIV at the vessel region fades and becomes low (red). However, the high LIV appearance recurred at later time points. At the 36 h time point, the vessel region shows the high LIV (green) again. At very late time points, e.g., at 84 h, the high LIV at the vasculature diminishes again. This oscillatory appearance of the LIV at the vessel was also observed in another healthy mouse liver sample under the same measurement protocol. Since we obtained similar results with Fig. 4 in another time-course study of healthy liver, we can therefore confirm that the result of high LIV appearance after 24 h might be repeatable. The reappearance of the high LIV at the liver microvascular complex might be related to some physiological process and its possible interpretation is described in the Discussion section.

**Figure 5:**
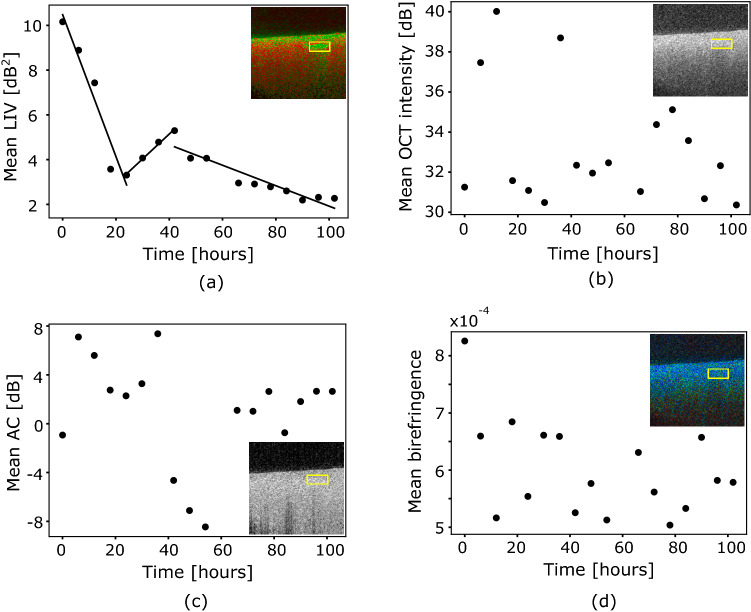
Time-course plots of mean (**a**) LIV, (**b**) OCT intensity, (**c**) attenuation coefficient (AC), and (**d**) birefringence of healthy mouse liver. The mean values of each quantity are computed within the region indicated by the yellow rectangular box shown in the insets of (**a**–**d**).

Note here that although the vessel was not clearly identified in the OCT signal at the later time points, it was visualized clearly in the LIV images.

The high LIV signal that we observed at the tissue surface at certain time points, e.g., at the 6 h and 24 h points, may be an artifact; such artifacts can be caused by dissociation of the liver tissue into the cultured medium. The dissociated liver tissue would form floaters in the cultured medium, and this may account for the high LIV (green) appearance of the medium at the later time points.

In the early time point (0 h), the vessel lumen diameter was quantitatively determined as 212 μm, while it changed to 112.5 μm at the later time point (36 h). This observed change in the lumen diameter would be due to contraction of the sample volume. Since the vessel lumen can not be observed clearly in OCT signal intensity at other time points (6 h, 12 h, 36 h, and 84 h), the lumen area diameter can not be quantified at these time points.

To observe the hourly changes in the tissue behavior in the microvascular complex of the healthy mouse liver in a more quantitative manner, mean values of the dB-scale OCT intensity, AC, LIV, and birefringence are plotted as a function of time, as shown in Fig. 5. The region of interest is indicated by the rectangular box shown in the inset of each part of Fig. 5, where the analysis region consists of 1548 pixels. The mean LIV result reveals three long-time-course tissue alteration phases in the microvascular complex (Fig. 5a). In the first phase, the mean LIV decreases monotonically with time until the 24 h time point. In the second phase, the mean LIV increases and reaches its highest value at the 42 h time point. In the third phase, the mean LIV value again decreases gradually over time.

The mean dB-scale OCT intensity (Fig. 5b) and the mean birefringence (Fig. 5d) do not show any clear tendencies. The mean AC (Fig. 5c) decreases over the period from 6 to 54 h, except for some outliers. It then became moderately high and reached a constant value at the later time points.

### Imaging of early fibrotic mouse liver at the initial time point

**Figure 6:**
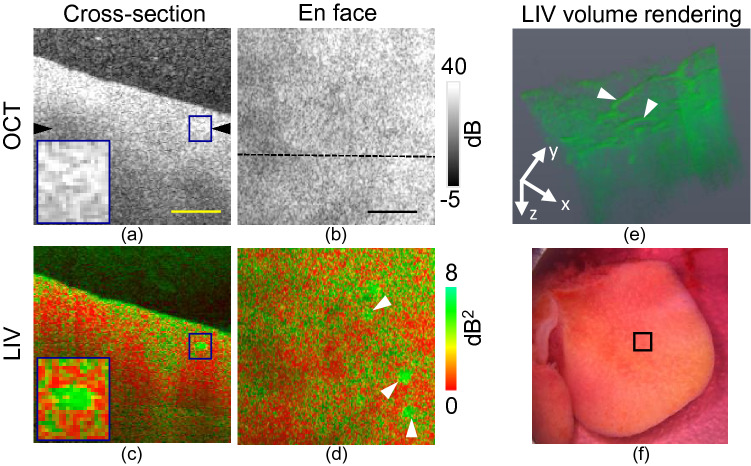
Dynamics imaging of early fibrotic mouse liver at the initial (0 h) time point. Cross-sections of (**a**) scattering OCT and (**c**) LIV images. (**b**, **d**) *En face* slices of the OCT and LIV images at the depth location indicated by black arrowhead in (**a**). (**e**) LIV volume rendering image and (**f**) original photograph of the measurement sample and the measurement area [square box]. Scale bar: 250 μm.

Figure 6 summarizes the results for the CCl_4_-treated early fibrotic mouse liver at the 0 h time point. Figure 6a and b represent the cross-sectional and *en face* images of scattering OCT, while Fig. 6c and d are those of the LIV. The locations of the *en face* slices and the B-scans are indicated by the black arrowhead shown in Fig. 6a and the horizontal line shown in Fig. 6b.

At the 0 h time point, we do not observe any lumen structure in the OCT intensity such as that shown in the healthy liver case at the same time point (Fig. 1). There is an isolated high LIV (green) spot in the cross-sectional LIV image, as indicated by a box and magnified in the inset shown in Fig. 6c; however, this spot was not observed in the OCT intensity image (Fig. 6a). These high LIV spots are also found in the *en face* LIV image (indicated by arrowheads in Fig. 6d). According to the volume rendering of the LIV image (Fig. 6e), these spots represent the cross-section of a vessel-like structure. It is notable that this vessel structure does not appear in the OCT scattering image, even at the initial time point, although it is seen clearly in the LIV image.

### Time-lapse imaging of early fibrotic liver for longitudinal evaluation

**Figure 7:**
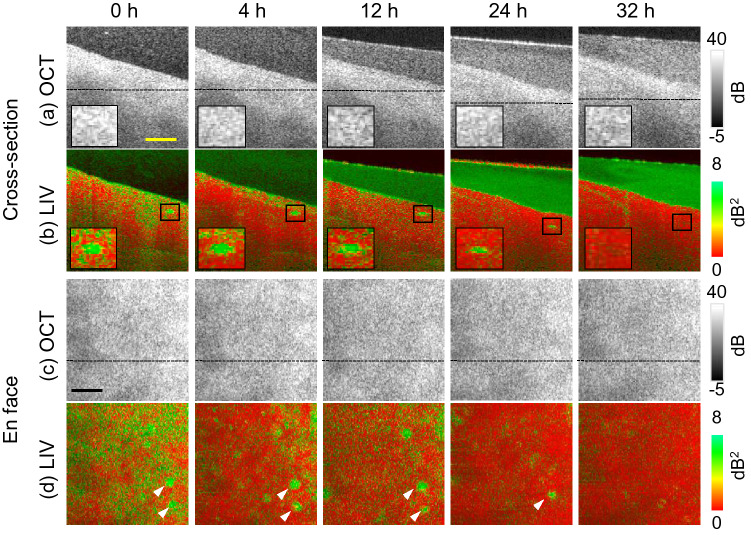
Longitudinal dynamics visualization of early fibrotic liver microvasculature. The first and second rows (**a**, **b**) represent the cross-sectional OCT and LIV images, whereas the third and fourth rows (**c**, **d**) show the *en face* OCT and LIV images, respectively. The first to fourth rows show time-course images at the 4, 12, 24, and 32 h time points. Scale bar: 250 μm.

Figure 7 shows the longitudinal time-course imaging of the early fibrotic liver at the 0, 4, 12, 24, and 32 h time points. Note here that the 0 h time point images are identical to those in Fig. 6. The first and second rows show cross-sectional images, while the third and fourth rows show *en face* images of the OCT intensity and the LIV. The depth locations of the *en face* slices are indicated in the corresponding cross-sectional images using black lines.

The LIV images (second and fourth rows) show that the OCT signal fluctuation magnitude is high (green) in the vessel region at the early time points, as shown in the enlarged images in the insets of Fig. 7b and indicated by the white arrowheads shown in Fig. 7d. However, the magnitude diminishes at the later time points. The medium at the later time points shows high LIV because of the small floaters in the cultured medium (Fig. 7b).

**Figure 8:**
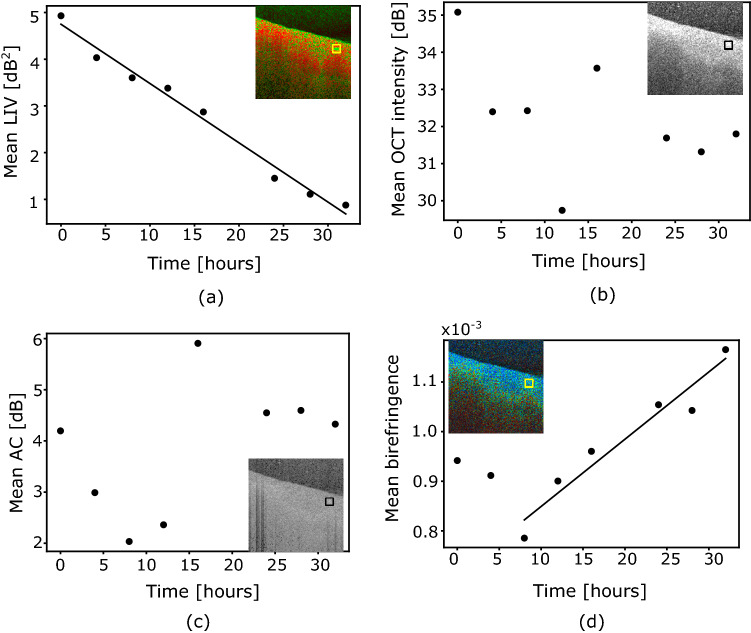
Time-course plots of the mean (**a**) LIV, (**b**) OCT intensity, (**c**) attenuation coefficient (AC), and (**d**) birefringence of the early fibrotic mouse liver. The mean values for each quantity are computed within the regions indicated by the square boxes in the insets of (**a**–**d**).

The hourly alterations in the tissue behavior can be observed more quantitatively by plotting the mean values of the LIV, the scattering OCT intensity, the AC, and the birefringence over time, as shown in Fig. 8. The computation regions for these mean values are indicated by the boxes shown in the insets of Fig. 8. The lines represent linear regressions.

The mean LIV in the vasculature decreases monotonically over time (Fig. 8a). The mean OCT intensity (Fig. 8b) does not show any clear tendency. The mean AC (Fig. 8c) shows that the AC decreases up to the 12 h time points and it then becomes moderate and constant after the 20 h time point. The mean birefringence increases gradually from 8 to 32 h (Fig. 8d).

## Discussion

In recent years, several OCT-based tissue dynamics imaging methods have been demonstrated to visualize and quantitate the intracellular motion within the tissue which is tightly associated with the tissue function^25,28–31^ . Dynamic OCT based on time-domain full-field OCT was first demonstrated to visualize subcellular metabolic contrast in tissues by temporal analysis of interferometric signals^25^. Time-spectroscopic analysis of the OCT signal was also developed to image the functional property of living tissues^29,30^. Recently, our group also demonstrated the dynamics OCT technique which includes the temporal variance (LIV) and the decorrelation of OCT signal intensity^28^. All these methods are based on the same principle i.e. temporal analysis of the time-sequence OCT signal intensity. These methods are successfully implemented and validated against conventional imaging techniques such as histology and fluorescence microscopy. Therefore, we believe that the LIV signal presented in this paper might correspond to the physiological process of the liver and might be associated with the tissue function of the liver.

In principle, the variation of the OCT signal in repetitive B-scans can be also caused by blood flow, sample motion due to breathing or heartbeat, Brownian motion due to moving particles, and the intercellular motion in the tissue. Since this study was performed ex vivo, therefore the blood flow signal and the sample motion can be neglected here. Brownian motion due to moving particles typically consists of random fast motion (order of millisecond) and it can only be detected when the scanning is faster than the particle dynamics. In this paper, the LIV signal was computed with a relatively slow-scanning protocol over the time window (time separation between the first and last frame) of 6.55 s. Therefore, the source of the high LIV signal cannot be caused by the Brownian motion. The only possibility in the variation of OCT signal therefore remains due to the intercellular motion in the tissue or tissue dynamics which generally exhibits a slow motion.

The microvascular unit of the liver is the hepatic lobule, which consists of the portal triad (the portal vein, the bile duct, and the hepatic artery), the central veins, and the sinusoid arranged in hepatocytes. The liver lobule has three distinct zones: the periportal zone (commonly known as zone 1), the intermediary zone (zone 2), and the perivenous zone (zone 3)^51^. In zone 3 (i.e., the perivenous zone), glycolysis linked to lipogenesis and Wnt signaling occurs more strongly than in the other zones^52–54^. In addition, the perivenous enzyme, i.e., glutamine synthetase, is known to be highly metabolically active^55^. In zone 1 (the periportal zone), the hepatocytes are more devoted to oxidative energy metabolism, gluconeogenesis, and urea synthesis than those in the perivenous zone^52^. The periportal hepatocytes contain enzymes for the tricarboxylic acid cycle and the respiratory chain. Lamers et al. reported that periportal enzyme such as carbamoylphosphate synthetase has high metabolic activity in human and rat liver acini^55^. In addition, similar to zone 3, equivalent pathways of Wnt signaling such as Ras/MAPK/ERK signaling are also highly occurred in zone 1^56^. Therefore, the periportal and perivenous zones, i.e., zones 1 and 3, are highly metabolically active.

Immediately after sacrification, a distinctly high LIV appeared around the bottom of the vessels in the healthy mouse liver (Fig. 1). This may indicate high metabolic activity at the periportal or perivenous zones. It should be noted here that the high LIV signature is only visible in deeper region or beneath the vessel. This asymmetric appearance of the dynamics around the vessel is worthy of further investigation in the future.

By performing a time-course study on mouse livers, Varmazyar et al. observed an increase in oxidative stress after 24 h after the liver was exposed to 2-chloroethyl ethyl sulphide-induced toxicity^57^. Oxidative stress is the mechanism that is involved during the imbalance between production and clearance of the reactive oxygen species (ROS) that induce cell and tissue damage. Several antioxidant enzymatic activities involving superoxide dismutase (SOD), catalase (CAT), glutathione reductase (GR), glutathione peroxidase (GPX), and glutathione S-transferase (GST) are involved in balancing ROS production and elimination^58^. In another study, hepatic regeneration due to cadmium-induced toxic injury was investigated in rat liver tissue ^59^. Based on the enzymatic activity of thymidine kinase (TK), liver regeneration with dual peak appearances at 12 and 48 h was found after the initial damage caused by toxicity and following hepatocellular death. Therefore, the liver tissue can be active posterior to it is damaged.

In our time-lapse imaging of the healthy liver (Figs. 4 and 5), it is natural for the metabolic activity around the liver microvasculature wall to decrease over time because this study is performed ex vivo. However, we observed a reappearance of the high LIV peak at 36 h. Although the hepatic regeneration behavior in the liver models described in the previous paragraph differs from our study, the enzymatic activity is a good candidate to explain the time course behavior of the liver dynamics observed in this study.

By comparing the mean values of the LIV, birefringence, and AC between the healthy and early fibrotic livers that were plotted as a function of time (see Fig. S2 in Supplementary File 2), we can find that just after the sacrification (0 h), the mean LIV signal at the microvascular complex was higher in the healthy liver than the fibrotic one. In addition, the reappearance of the high LIV signal was observed in the healthy liver which was not found in the fibrotic liver. The mean birefringence of the fibrotic liver at the microvascular complex was larger than the healthy liver at the 0 h time point. And, there is no such significant difference observed in AC contrasts between the two livers.

The mean birefringence around the microvascular complex at the 0 h time point was higher in the CCl_4_-treated fibrotic liver (Fig. 8d) than in the normal liver (Fig. 5d). Klaas et al.^60^ reported the presence of extracellular matrix structural components, including type-IV collagen, around the periportal region of a healthy mouse liver and also reported an increase in the collagen content in the CCl_4_-injected mouse liver. Collagenous tissues are known to be birefringent^61^, and thus a higher collagen density would cause higher birefringence. Therefore, the higher birefringence in the CCl_4_-treated fibrotic liver might indicate a higher collagen density than the normal liver.

Time lapse imaging result for the birefringence show that the mean birefringence of the early fibrotic liver around the microvascular complex increases over time (Fig. 8d). In previous PS-OCT studies, it was found that the phase retardation, which is proportional to the birefringence, increases when the collagen density increases^62^. During the longitudinal ex vivo measurement process, we did observe liver tissue shrinkage. Therefore, this increase in the birefringence can at least be partially accounted for by the increased collagen density due to this shrinkage.

The attenuation coefficient is a combination of the absorption and scattering coefficients of the tissue^36,63^. However, in rodent livers, the attenuation coefficient is mainly dominated by the scattering coefficient rather than the absorption coefficient^64^. Because higher tissue density would cause higher scattering, the attenuation coefficient thus represents the tissue density. Therefore, an increase or reduction in the mean attenuation coefficient at the microvascular complex (Figs. 5c and 8c) can be interpreted as either an increase or reduction of the tissue density caused by shrinkage or swelling, respectively.

In our study, the OCT images were not actively used to investigate the tissue property. However, the OCT images are important to identify the landmark tissues. The cross-sectional and *en face* OCT intensity present the advantage that it can visualize the vessel (lumen) with high clarity just after the sacrification (Fig. 1). Without such visualization, our hypothesis, the high LIV signal in the mouse is associated with the high activity at the vessel surrounding tissue, which has been discussed earlier, had not been proved. In addition, by utilizing the OCT signal intensity, we quantitatively determine the vessel diameter change over time.

To evaluate the interrelation between the OCT signal intensity, the AC, the birefringence, and the LIV, the correlation coefficients among them are summarized in Table 1. For this analysis, the mean, standard deviation, maximum, and minimum values of the correlation coefficients were computed over all the time points of the healthy and early fibrotic livers. For the healthy liver, the analysis region is shown in the insets of Fig. 5 and this region consists of 1548 points (i.e., pixels). The analysis region for the fibrotic liver is shown in the insets of Fig. 8 and this region consists of 1600 points. No significant correlation was observed among the features, except between the OCT and the AC. The correlation between the OCT and the AC is unimportant because both the OCT and the AC are about the scattering properties of the tissue and the AC is even computed directly from the OCT. Moderate correlation was observed between OCT and LIV in healthy liver. These low correlations between the birefringence, the LIV, and the AC indicated that this multi-contrast measurement is not redundant.

**Table 1:** Mean, standard deviation (SD), maximum (Max), and minimum (Min) values of the correlation coefficients between multiple contrasts including OCT, LIV, AC, and birefringence of the healthy and early fibrotic livers over all the time points.

	Healthy liver		Fibrotic liver	
	Mean ± SD	[Max, min]	Mean ± SD	[Max, min]
OCT vs LIV	− 0.54 ± 0.17	[− 0.10, − 0.68]	− 0.38 ± 0.16	[− 0.16, − 0.64]
OCT vs AC	0.90 ± 0.06	[0.97, 0.76]	0.93 ± 0.02	[0.96, 0.88]
OCT vs BR	− 0.31 ± 0.04	[− 0.25, − 0.44]	− 0.36 ± 0.03	[− 0.32, − 0.41]
LIV vs AC	− 0.54 ± 0.15	[− 0.12, − 0.66]	− 0.39 ± 0.16	[− 0.16, − 0.64]
LIV vs BR	0.16 ± 0.05	[0.27, 0.10]	0.17 ± 0.04	[0.24, 0.13]
AC vs BR	− 0.29 ± 0.05	[− 0.21, − 0.42]	− 0.33 ± 0.04	[− 0.27, − 0.41]

*AC* attenuation coefficient, *LIV* log-intensity variance, *BR* Birefringence.

The feature score plot for the principal component analysis (Fig. 2b) suggests that the attenuation coefficient, which represents the scattering coefficient property of the tissue, is independent of the other two contrasts, i.e., the LIV and the birefringence. The LIV and the birefringence make opposite relative contributions to PC1 and PC3. This suggests that the LIV and the birefringence can provide nonredundant information required for tissue discrimination. The attenuation can be used solely to discriminate tissue types, whereas the LIV and the birefringence are better when used simultaneously to discriminate the tissue status.

In the Pseudo-color composite PC image of healthy liver, we observed two distinct zones (Fig. 3). These two zones are termed as zone 1 and zone 2, respectively . Zone 1 is referred to as the moderate depth region (0 to 0.46 mm) which appeared as green, while zone 2 is referred to as the deep tissue region (0.46 to 1 mm) which appeared as red in the pseudo color composite PC image. PC3 dominates zone 1, while PC1 dominates zone 2. In PC1, LIV and birefringence collocate with the same magnitude and in PC3, LIV and birefringence antilocate with each other (Fig. 2b). Since LIV is sensitive to tissue activity and birefringence is sensitive to fibrotic tissue therefore, zone 1 can be interpreted as active-non fibrotic tissue type, while zone 2 can be interpreted as active-fibrotic tissue.

## Conclusion

We have investigated tissue activity in a label-free manner around the microvasculature of healthy and early fibrotic mouse livers using a newly developed OCT-based dynamics imaging method and a custom-built JM-OCT system. We visualized high dynamics successfully beneath the vessel region of healthy mouse livers and several isolated high dynamics spots in early fibrotic model livers just after the dissection of mice. Furthermore, via longitudinal imaging of the mouse livers, we demonstrated the degradation of the tissue activity quantitatively and also monitored the detailed long-time-course changes in the microstructural properties using the attenuation coefficient and the local birefringence. We also applied principal component analysis to the multi-contrast signals acquired using the JM-OCT system to discriminate the different tissue types. In conclusion, the multi-contrast imaging technique based on JM-OCT could be feasible for longitudinal visualization and monitoring of functional and structural changes in liver microvasculature complex. This label-free and non-destructive imaging method could add additional insights to the current standard imaging modalities.

## Methods

### Ex vivo mice liver model and study protocol

To perform imaging of the functional and structural properties of the liver microvasculature, two different sample types have been used in this study. The first type is healthy mouse livers and the second is early fibrotic mouse model livers induced by CCl_4_ injection^65^. Ex vivo experimental studies were performed in this work. Wild type C57BL/6 mice at an age of 5-7 weeks were used in the experiments.

For the healthy liver study, the mice were sacrificed by cervical dislocation to extract their livers. The mouse liver was immersed in a cultured medium (Dulbecco’s modified eagle medium (DMEM)) immediately after dissection to avoid quick death of the tissues. After 1 h, at a time denoted as the initial or zero-hour time point, measurements were performed using the JM-OCT system at room temperature (24 °C). Longitudinal measurement was performed every 6 h for a period of up to four days.

For the early fibrotic liver model, an acute liver injury was induced by intraperitoneal injection of CCl_4_ once per day for two consecutive days and the mice were then sacrificed by cervical dislocation after 48 h. Similar to the healthy liver study, the early fibrotic liver was also immersed in a DMEM cultured medium just after sacrification and was then kept at room temperature (24 °C). After 1 h, i.e., at the initial or zero-hour time point, the first OCT measurement was performed. The longitudinal measurement was performed every 4 h until the 32 h time point.

Note that, for the two longitudinal studies, we imaged the healthy and early fibrotic livers after every 6 h and 4 h respectively. According to our pilot study, the alteration of the LIV signal over time in the healthy liver is slow and it continues for more than four days. On the other hand, that of fibrotic liver was faster than the healthy liver and it persisted for less than two days. So, we optimized the measurement interval and measurement duration independently for these two cases.

All animal experiments were performed in accordance with the animal study guidelines of the University of Tsukuba. All experimental protocols involving mice were approved by the Institutional Animal Care and Use Committee (IACUC) of University of Tsukuba. The present study was designed, performed, and reported according to the principles of ARRIVE (Animal Research: Reporting of In vivo Experiments) guidelines.

### Jones matrix OCT system and data acquisition protocol

A custom-built Jones-matrix-based PS-OCT (JM-OCT) system was used in this study^49,50^. The system uses a microelectromechanical system (MEMS)-based wavelength sweeping laser source (AXP50124-8, Axun Technologies, MA ) with a central wavelength of 1310 nm and a scanning rate of 50 kHz. The power incident on the sample is approximately 14 mW and the system sensitivity is 104 dB. The lateral resolution (1/e^2^-width) is 19 μm and the lateral pixel separation is 1.95 μm. The axial (depth) resolution (full width at half-maximum) was 19 μm in air (14 μm in the tissue), while the depth-pixel separation was 10 μm in air (7.24 μm in the tissue).

In comparison to a standard OCT device, the JM-OCT has two additional hardware modules, comprising a polarization delay unit and a polarization diversity detector. Because it includes these two modules, the JM-OCT system provides four complex OCT signals corresponding to the four polarization channels that then form a Jones matrix of the sample. The local phase retardation or birefringence is computed from the Jones matrix. A more detailed description of the system can be found elsewhere in the literature^49,50^. The JM-OCT measures multi-contrast images simultaneously, including backscattering intensity, LIV, attenuation coefficient, and birefringence images.

To perform volumetric imaging, the JM-OCT system captures 16 frames at a single location and 128 locations on the sample have been scanned. At each location, the time separation between the first and last frames, which we call the time window, is 6.55 s. The scan is a raster scan with 512 A-lines and a total of 2048 frames are obtained. The total acquisition time for a volume is 26.2 s.

### Logarithmic intensity variance (LIV) imaging

To compute the LIV image, we first computed a polarization-insensitive OCT intensity image by averaging the absolute-squared intensities of four Jones matrix entries, i.e., four OCT signals corresponding to the four polarization channels. The pixel-by-pixel depth-resolved LIV image is then computed as the variance of the time-sequence logarithmic (dB) scale OCT intensity signal at each pixel^28^

1 $$\begin{aligned} \text {LIV}(x,z) = \frac{1}{N}\sum _{i = 0}^{N-1} \left[ I_{dB}\left( x,z,t_i\right) - \left\langle {I_{dB}(x,z)}\right\rangle _{t_i} \right] ^2 \,, \end{aligned}$$

where ${I_{dB}}$ is the dB-scale OCT intensity, *x* and *z* are the lateral and axial positions, respectively, $t_i$ is the acquisition time point of the *i*-th frame, $\left\langle {I_{dB}(x,z)}\right\rangle _{t_i}$ is the averaged dB-scale OCT intensity over $t_i$, and *N* is the number of frames. It is evident from Eq. (1) that the LIV is sensitive to the magnitude of the OCT signal fluctuation.

To enhance the readability of the dynamics image, a pseudo-color composition is created by combining the dynamics image, the LIV and the OCT intensity image , as described in Section 2.3 of Ref.^28^. In this composition, the color hue (H channel) is the LIV, the pixel brightness (V channel) is the dB-scale OCT intensity (V channel), and the saturation (S channel) is set at 1 for all pixels. In the pseudo-color composite image, the high dynamics region appears in green, while the low dynamics region appears in red.

The LIV images are shown not only as 2D sections but also are shown by volume rendering. Before the volume rendered image is generated, the tissue surface is first segmented using the dB-scale OCT intensity signal, and the medium above the tissue surface is excluded. The low intensity regions are then excluded from the tissue region using an intensity threshold mask. The LIV image is then binarized using a threshold. The threshold value in this case was 10 dB^2^, which was selected to mask the LIV image appropriately. If the LIV of the target pixel was higher than this threshold, a value of 1 was assigned; otherwise, a value of 0 was assigned. Finally, the processing was performed frame-by-frame for the entire 3D dataset to obtain a final 3D LIV data volume that represented the high LIV regions only.

### Attenuation coefficient (AC) imaging

To compute the AC, the scattering OCT is obtained by averaging the absolute squared intensities of the four Jones matrix tomographies corresponding to the four polarization channels. The pixel-by-pixel depth-resolved AC is then computed using Eq. (18) from Ref.^36^.

We consider the AC to mainly represent the local scattering properties of the tissue because this study was originally performed on ex vivo liver tissues, in which the scattering coefficient is more dominant when compared with the absorption in the tissue within the measured wavelength^64^.

### Birefringence imaging

The sample birefringence or equivalent local phase retardation is computed using the local Jones matrix analysis method^40^. The local (depth-localized) Jones matrix was obtained from two matrices with 8-pixel (57.9 μm in the tissue) depth separation. Two eigenvalues of the local Jones matrix are computed and the local phase retardation is then obtained as the phase difference between these two eigenvalues. The local phase retardation is then scaled linearly to obtain the birefringence using Eq. (2) from Ref.^66^. The birefringence is then processed further using a maximum *a-posteriori* (MAP) birefringence estimator^66^ with a 2 × 2 pixel spatial kernel to enhance the accuracy.

For enhanced visualization, a pseudo-color composite image is created by combining the OCT intensity with the birefringence and the estimation reliability, as described in Section 3.4 of Ref.^66^. Here, the pixel brightness, the color hue, and the saturation represent the OCT intensity, the birefringence, and the reliability, respectively.

### Principal component analysis (PCA)

The JM-OCT system provides multi-contrast images that include the LIV, AC, and birefringence, where each contrast represents different tissue properties. These multi-contrast images can be combined to synthesize tissue-specific contrasts for improved tissue discrimination and better tissue structure visualization. To generate a synthesized tissue contrast, PCA has been performed using the three input contrasts mentioned above.

Before performing PCA, the tissue surface was first segmented automatically, as described in Ref.^50^. An intensity threshold mask was then applied to remove the low signal region, and the region ranging from the tissue surface to a 100-pixel (724-μm) depth was then processed by PCA.

The PCA was applied to the volumetric data and computed three principal components (PCs) from the three contrasts. Three volumetric tomographies of the PCs were also computed. The PCA was performed using a machine learning library (scikit-learn 0.18 on Python 3.8).

The PCs of an input matrix are the eigenvector-eigenvalue pairs of its covariance matrix. The first to last PCs in order will have the highest to lowest eigenvalues. Similarly, the corresponding eigenvectors will have variances ranging from the highest to the lowest. The feature score of a PC is given by the eigenvectors, for which the entries represent the loading of each original contrast into the PC.

The pseudo-color composite PC image is then generated by assigning the red, blue, and green channels to PC1, PC2, and PC3, respectively.

## Supplementary Information

Supplementary Information 1.

Supplementary Information 2.
