# Supplementary material for: Label-free functional and structural imaging of liver microvascular complex in mice by Jones matrix optical coherence tomography
Source: Sci Rep. 2021 Oct 8;11:20054. doi: 10.1038/s41598-021-98909-6 (PMC8501041; doi:10.1038/s41598-021-98909-6)
Supplement: Supplementary file 1 — Supplementary Information 1. [file 41598_2021_98909_MOESM1_ESM.pdf]

Supplementary material 1

Label-free functional and structural imaging of liver microvascular complex in mice by Jones matrix optical coherence tomography

Pradipta Mukherjee[1], Arata Miyazawa[1], Shinichi Fukuda[2,3], Toshiharu Yamashita[4], Donny Lukmanto[3], Kosuke Okada[5], Ibrahim Abd El-Sadek[1,6], Lida Zhu[1], Shuichi Makita[1], Tetsuro Oshika[2], and Yoshiaki Yasuno[1]

[1]*Computational Optics Group, University of Tsukuba, Tsukuba, Ibaraki, Japan*
[2]*Department of Ophthalmology, Faculty of Medicine, University of Tsukuba, Tsukuba, Ibaraki, Japan*
[3]*Department of Advanced Vision Science, Faculty of Medicine, University of Tsukuba, Tsukuba, Ibaraki, Japan*
[4]*Laboratory of Regenerative Medicine and Stem Cell Biology, Graduate School of Comprehensive Human Sciences, University of Tsukuba, Tsukuba, Ibaraki, Japan*
[5]*Divison of Medical Sciences, Faculty of Medicine, University of Tsukuba, Tsukuba, Ibaraki, Japan*
[6]*Department of Physics, Faculty of Science, Damietta University, 34517 New Damietta City, Damietta, Egypt*

Figure S1 summarizes cross-sectional OCT intensity and LIV images of four different healthy mouse liver samples at the initial time point (0-hour). In cross-sectional OCT images, the lumen appears as hyposcattering region [arrows, Fig. S1(a)]. This low scattering region is identical to Fig. 1(a) which corresponds to the vessel. Cross-sectional LIV images show high temporal fluctuation of OCT signal just beneath the lumen which is shown by arrowheads in Fig. S1(b). These high LIV signals are also consistent with Fig. 1(e).

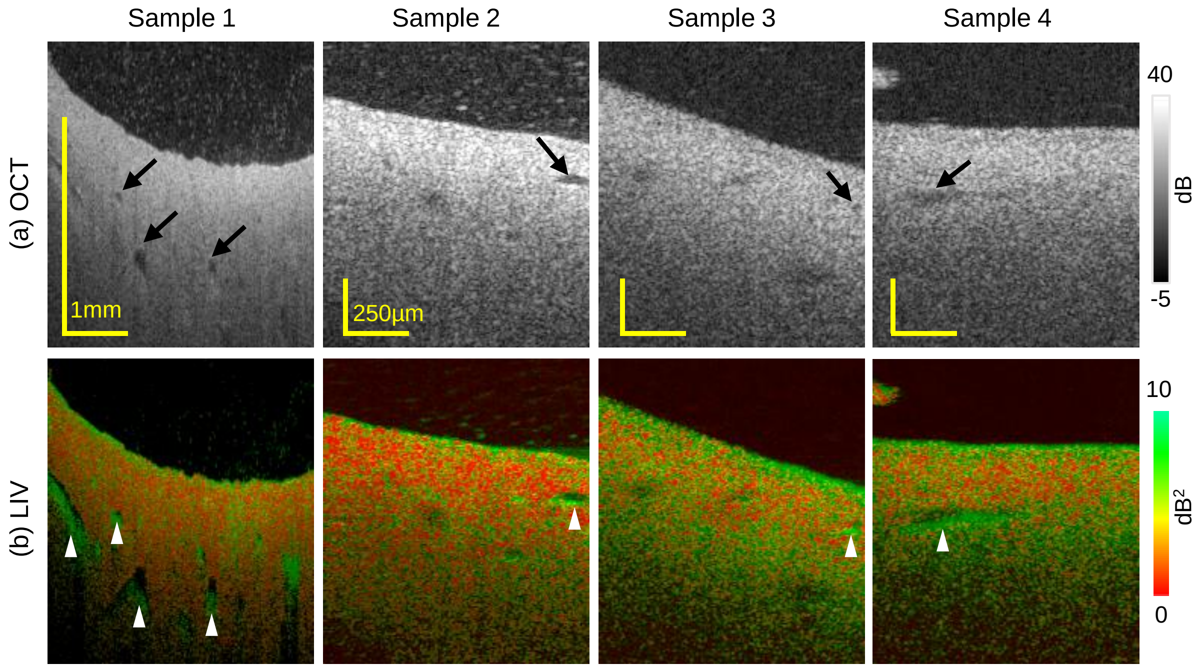

Figure S1: Cross-sectional OCT (first row) and LIV (second row) imaging of four different healthy mouse livers at initial time point (after 1 hour of sacrification).
