# Supplementary material for: Label-free functional and structural imaging of liver microvascular complex in mice by Jones matrix optical coherence tomography
Source: Sci Rep. 2021 Oct 8;11:20054. doi: 10.1038/s41598-021-98909-6 (PMC8501041; doi:10.1038/s41598-021-98909-6)
Supplement: Supplementary file 2 — Supplementary Information 2. [file 41598_2021_98909_MOESM2_ESM.pdf]

Supplementary material 2

Label-free functional and structural imaging of liver microvascular complex in mice by Jones matrix optical coherence tomography

Pradipta Mukherjee[1], Arata Miyazawa[1], Shinichi Fukuda[2,3], Toshiharu Yamashita[4], Donny Lukmanto[3], Kosuke Okada[5], Ibrahim Abd El-Sadek[1,6], Lida Zhu[1], Shuichi Makita[1], Tetsuro Oshika[2], and Yoshiaki Yasuno[1]

[1]*Computational Optics Group, University of Tsukuba, Tsukuba, Ibaraki, Japan*
[2]*Department of Ophthalmology, Faculty of Medicine, University of Tsukuba, Tsukuba, Ibaraki, Japan*
[3]*Department of Advanced Vision Science, Faculty of Medicine, University of Tsukuba, Tsukuba, Ibaraki, Japan*
[4]*Laboratory of Regenerative Medicine and Stem Cell Biology, Graduate School of Comprehensive Human Sciences, University of Tsukuba, Tsukuba, Ibaraki, Japan*
[5]*Divison of Medical Sciences, Faculty of Medicine, University of Tsukuba, Tsukuba, Ibaraki, Japan*
[6]*Department of Physics, Faculty of Science, Damietta University, 34517 New Damietta City, Damietta, Egypt*

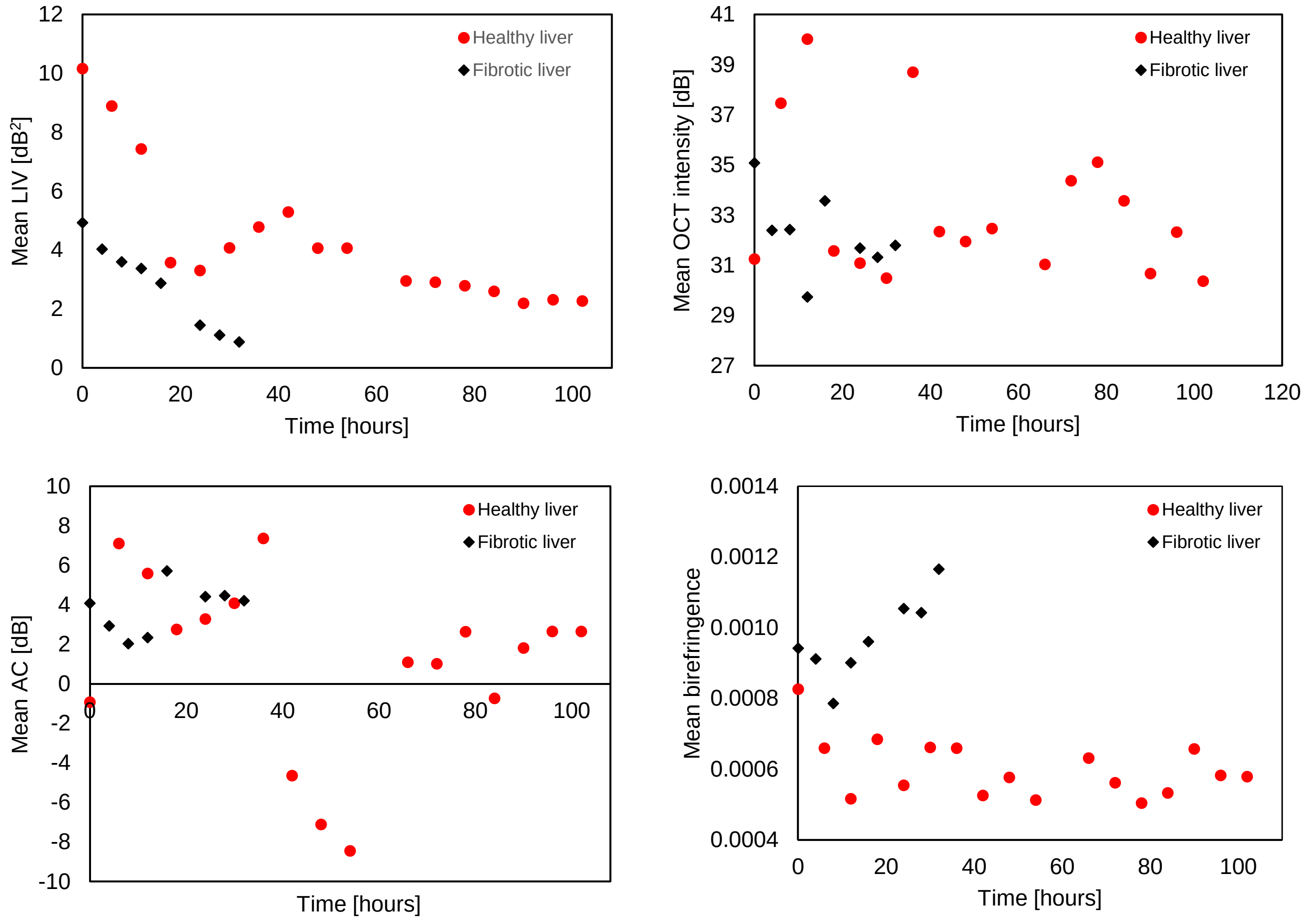

Figure S2: Comparison of mean LIV, OCT intensity, AC, and birefringence between healthy and fibrotic mouse livers.
